# Alcohol use in late adolescence and early adulthood: The role of generalized anxiety disorder and drinking to cope motives

**DOI:** 10.1016/j.drugalcdep.2019.04.044

**Published:** 2019-11-01

**Authors:** Maddy L. Dyer, Jon Heron, Matthew Hickman, Marcus R. Munafò

**Affiliations:** a School of Psychological Science, University of Bristol, Bristol, United Kingdom; b Population Health Sciences, Bristol Medical School, University of Bristol, Bristol, United Kingdom; c MRC Integrative Epidemiology Unit (IEU), University of Bristol, Bristol, United Kingdom

**Keywords:** Generalized anxiety disorder, Alcohol, Drinking-To-Cope, ALSPAC, Longitudinal

## Abstract

**Background:**

The relationship between anxiety and alcohol use is unclear, and moderating factors, such as drinking to cope (DTC) motives, may explain mixed findings.

**Methods:**

Using the Avon Longitudinal Study of Parents and Children (ALSPAC), we examined associations between generalized anxiety disorder (GAD) at age 18 and frequent drinking, frequent bingeing, hazardous drinking, and harmful drinking at ages 18 (unadjusted n = 3462) and 21 (unadjusted n = 2076), in a sample of late adolescent drinkers. Analyses were adjusted for sociodemographic, parental, and adolescent confounders. We also examined whether DTC motives influenced the strength and direction of associations between GAD and alcohol use.

**Results:**

GAD was positively associated with all alcohol outcomes at baseline (unadjusted OR (95% CI): frequent drinking 1.40 (1.02–1.91); frequent bingeing 1.40 (0.96–2.04); hazardous drinking 1.44 (1.08–1.92); harmful drinking 1.98 (1.22–3.23)). GAD increased the odds of harmful drinking at age 21 (unadjusted OR 1.72, 95% CI 1.09–2.73), but there was no clear evidence of a longitudinal relationship between GAD and the other alcohol use outcomes. There was no clear evidence of a GAD x DTC interaction on alcohol use at ages 18 or 21. Findings were consistent across various multiply imputed datasets.

**Conclusions:**

In adolescence, GAD symptoms are associated with frequent drinking, frequent bingeing, hazardous drinking, and harmful drinking. In early adulthood, associations remain for harmful drinking only. DTC motives do not appear to moderate the relationship at either age.

## Highlights

• Generalized anxiety is positively associated with alcohol use in adolescence.
• In early adulthood, associations remain for harmful drinking only.
• Associations are not moderated by drinking to cope motives.

## 1 Introduction

Substance use disorders, particularly alcohol abuse and dependence, are the most common psychiatric disorders in adolescence (12%), followed by anxiety disorders (11%) (Costello et al., 2011). Anxiety and alcohol disorders frequently co-occur (Smith and Randall, 2012), and this comorbidity is associated with poorer recovery compared to each condition individually (Bruce et al., 2005; Driessen et al., 2001). It is therefore important to determine the temporal sequence of associations between anxiety and alcohol use.

The self-medication hypothesis suggests anxious individuals may use alcohol to cope with their emotional distress and alleviate physical symptoms (Khantzian, 1990; Sher and Levenson, 1982). According to this hypothesis, anxiety is a risk factor for later alcohol problems (via negative reinforcement), which is supported by some longitudinal evidence from adolescent samples. For example, Frojd et al. (2011) found generalized anxiety at age 15 was associated with a higher incidence of frequent alcohol use two years later. However, other longitudinal studies have shown an inverse relationship. For example, Pardini et al. (2007) found adolescent boys with anxiety were less likely to develop alcohol use disorder symptoms 12 years later. Possible explanations for a protective effect of anxiety include social withdrawal and fear of negative consequences associated with risky drinking (Pardini et al., 2007). Several studies also have found no clear evidence of a prospective relationship between generalized anxiety in adolescence and subsequent alcohol use (Marmorstein, 2015), or alcohol use disorders (Abram et al., 2015; Wolitzky-Taylor et al., 2012; Zimmermann et al., 2003).

The mixed evidence may be explained by other factors influencing the strength and direction of the anxiety-alcohol relationship; anxiety could act as a risk or protective factor if there are moderating influences. One factor that may moderate this relationship is drinking to cope (DTC), the tendency to drink alcohol to relax, forget worries, cheer up, cope with depression or nervousness, or to feel more self-confident (Cooper et al., 1992). Higher anxiety is associated with greater DTC (Stapinski et al., 2016), and DTC motives are a risk factor for later alcohol problems (Kuntsche et al., 2005) and dependence (Crum et al., 2013). There is some evidence that DTC moderates the relationship between anxiety and alcohol problems in adult samples. For example, in one study, people with an anxiety disorder who self-medicated with alcohol were more likely to have an additional alcohol use disorder three years later compared to anxious individuals who did not self-medicate (Menary et al., 2011). Other research has provided cross-sectional evidence for an interaction between anxiety and DTC motives in an adolescent sample. Higher anxiety symptoms were associated with greater alcohol problems among individuals with high DTC motives but not those with low DTC motives (Goldstein et al., 2012). Although this study was conducted with a high-risk sample (adolescents involved with child welfare).

In the current study, we investigated whether generalized anxiety disorder (GAD) at age 18 was associated with frequent drinking, frequent bingeing, hazardous drinking, and harmful drinking at baseline and longitudinally at age 21 and we tested whether adolescent DTC motives moderated these associations. In both the cross-sectional and longitudinal analyses, we hypothesized that: (a) GAD would be positively associated with all alcohol outcomes, and (b) the strength of associations would be greater in those who also endorse high (vs. low) DTC motives.

## 2 Material and methods

### 2.1 Participants

We used data from the Avon Longitudinal Study of Parents and Children (ALSPAC), a prospective, population-based birth cohort study (Boyd et al., 2013; Fraser et al., 2013). A total of 14,541 pregnant women living in the former Avon Health Authority, with expected delivery dates between April 1 st, 1991 and December 31 st, 1992, were recruited into the study (http://www.bristol.ac.uk/alspac/). Data has been collected on the core participants, their mothers, fathers, grandparents, siblings, and now their offspring via questionnaires and focus clinics. Of the 13,978 singletons/twin offspring alive at one year, a small number of participants have since withdrawn consent (n = 24) leaving a starting sample of 13,954. In the late 1990’s an attempt was made to bolster the sample by recruiting additional eligible participants. Here we focus on the ‘core’ cases from phase 1 recruitment and exclude these later enrollers due to their lack of early data.

The study website contains details of all the data that is available through a fully searchable data dictionary (http://www.bris.ac.uk/alspac/researchers/data-access/data-dictionary/). Ethics approval for the study was obtained from the ALSPAC Ethics and Law Committee and the Local Research Ethics Committees.

We focused on data collected when the participants were age 18 years (median 17.8 years, IQR 17.6 to 17.9) and age 21 years (median 20.9 years, IQR 20.5 to 21.4). The age 18 baseline data were obtained from a subsample of the ALSPAC cohort who attended the ‘Teen Focus 4’ research clinic (n = 4878), while the age 21 follow-up data were collected via questionnaire which was administered either online or through the post (n = 3772).

### 2.2 Measures

#### 2.2.1 Generalized anxiety disorder (GAD)

GAD was assessed at age 18. Participants completed a self-administered computerized version of the Clinical Interview Schedule-Revised (CIS-R) (Lewis et al., 1992), which uses computer algorithms to identify psychiatric disorders according to DSM-IV and ICD-10 criteria. A binary variable indicating presence of GAD vs. no diagnosis was taken as our primary exposure measure with sensitivity analyses examining a variant in which participants with depression or other forms of anxiety were excluded from the reference group. Because different types of anxiety may have distinct associations with alcohol use, we decided not to derive a single variable to denote presence vs. absence of any anxiety disorder, as this amalgamation of anxiety variables may dilute any existing effects.

#### 2.2.2 Drinking to cope (DTC) motives

DTC motives were assessed at age 18. Participants completed a modified version of the Drinking Motives Questionnaire (Cooper et al., 1992), which has good internal consistency (α = 0.79) (Stapinski et al., 2016). The five original ‘coping’ items measured how often participants use alcohol to relax, forget worries, cheer up, cope with depression or nervousness, or feel more self-confident, over the past two years. Our adapted scale separates the “cope with depression or nervousness” item into two items, and an additional item was created (“drinking to help when your mood changes a lot”). Participants rated on a four-point ordinal scale how frequently they drink alcohol for each reason: 0 ‘almost never’, 1 ‘sometimes’, 2 ‘often’, 3 ‘almost always’. The seven items were summed, and the resulting scale was dichotomized at the top quartile.

#### 2.2.3 Alcohol use

Alcohol use was assessed at age 18 and 21 using the Alcohol Use Disorders Identification Test (AUDIT) (Babor et al., 2001). From this we derived four binary alcohol outcome variables: frequent drinking, frequent bingeing, hazardous drinking, and harmful drinking. Drinking alcohol ‘2 to 4 times a month’, ‘monthly or less’, or ‘never’, was coded as infrequent drinking. Drinking alcohol ‘2 to 3 times a week’, or ‘4 or more times a week’ was coded a frequent drinking. Individuals who consume six or more units on one occasion ‘monthly’, ‘less than monthly’ or ‘never’ were coded as infrequent binge drinkers, and those who consume six or more units ‘weekly’ or ‘daily or almost daily’ were coded as frequent binge drinkers. Individuals who scored ≥ 8 on the AUDIT were classified as hazardous drinkers, and scores of ≥ 16 indicated harmful drinking (Babor et al., 2001). We converted the original drinking frequency and bingeing frequency items from 5-level ordinal variables to binary variables, for consistency with the other two alcohol outcomes and for ease of interpretation.

The AUDIT is only of relevance to participants who have ever consumed alcohol, and in a clinical setting many of the questions would be skipped if the patient reported abstention during the last year. As being a non-drinker precludes the use of alcohol as a coping motive, we excluded individuals who had either never consumed alcohol or not consumed alcohol in the last year. As a sensitivity analysis, models which did not feature DTC were re-estimated whilst retaining the non-drinkers with these cases assigned a value of zero for each binary alcohol measure. Subsequent abstention from alcohol was permitted for the 21-year alcohol outcomes however there was only a handful of cases in this instance.

#### 2.2.4 Potential confounders

The following variables were included as potential confounders: sociodemographic variables (gender, maternal education, family income, housing tenure, and social class), parental variables (parental depression, anxiety, alcohol use, and tobacco use), and adolescent variables (tobacco use, cannabis use, drinking frequency and bingeing frequency four years earlier than the baseline alcohol outcomes, conduct problems, and emotional symptoms). Confounders were selected based on their *a priori* relevance and/or their associations with both anxiety and alcohol use in the literature. Supplementary Fig. 1 provides a timeline of all study variables.

### 2.3 Statistical analyses

All analyses were conducted in Stata 14. We used logistic regressions to examine the relationship between GAD at age 18 and frequent drinking, frequent bingeing, hazardous drinking and harmful drinking at ages 18 and 21. We assessed the impact of potential confounding by comparing unadjusted results (model 1) with results cumulatively adjusted for sociodemographic covariates (model 2), parental covariates (model 3), and adolescent covariates (model 4). In the prospective analyses, we did not adjust for baseline alcohol use as we thought this would result in model over-adjustment. We examined evidence of effect modification by conducting interaction tests (i.e., including a GAD × DTC interaction term), and then stratifying analyses by DTC motives (high vs. low). Regardless of the results of the interaction tests, we present all interaction analyses stratified for completeness in the supplementary material.

#### 2.3.1 Missing data

**Fig. 1:**
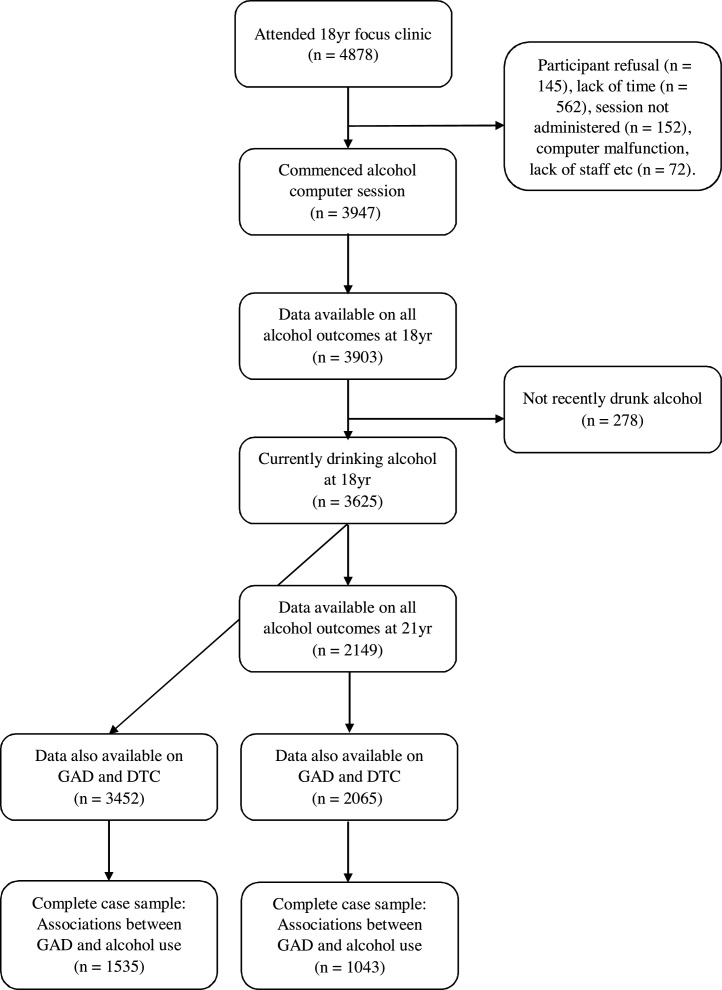
‘Study sample size flow diagram’. This figure shows a breakdown of how the final analysis samples were determined. Yr. = year; GAD = generalized anxiety disorder; DTC = drinking to cope.

A breakdown of how the final analysis samples were determined is shown in Fig. 1. Although 4878 young people attended the 18 yr clinic, only 3947 started the computer session which comprised questions on a range of behaviors including alcohol use, other substances and antisocial behavior. Whilst this sample-reduction from 4878 to 3947 is substantial, only a minority of cases were down to participant refusal (Fig. 1). Of the participants who started the computer session, 3903 provided responses the ten AUDIT questions, with 278 reporting that they had never or not recently drunk alcohol which left a sample of 3625 with all four baseline alcohol measures.

Initially models were estimated using all available data, however since 3625 represents a small proportion of those initially enrolled in ALSPAC, and the inclusion of confounders from a range of earlier questionnaires and clinics led to further reductions, we sought to examine the potential for non-random attrition leading to distorted conclusions. It is inevitable in longitudinal studies that loss-to-follow-up will be patterned in some way, and ALSPAC is no exception. However, socially-patterned dropout does not imply bias and an analysis based on available data will be unbiased provided the independent variables explain any systematic differences in the outcome between those included and excluded from the model. The use of multiple imputation increases the likelihood that a Missing At Random assumption can be made as auxiliary data can be included, and here we use the wealth of additional data in the ALSPAC resource in an attempt to sever any link between the model variables and the missingness mechanism.

A succession of multiply-imputed datasets was produced in order to examine the robustness of the available-data estimates. Firstly, 21-year alcohol and confounder information were predicted amongst the 3625 with baseline alcohol data and the results from these analyses are shown in the main document. Following this, the imputation sample was boosted to 4600 (imp#2) and then to 9278 (imp#3) to include those who attended the clinic but did not complete the alcohol session and those who were invited to the clinic but did not attend. For these imputations we made the simplifying but not wholly unreasonable assumption that all these additional cases would have been eligible to complete the whole AUDIT. Results from these imputed datasets can be found in the Supplementary material.

Imputation was carried out using multivariate imputation by chained equations, implemented using the -ice- command (Royston and White, 2011). Twenty cycles of regression switching were used for all imputation models. Both the quantity of auxiliary data and the number of datasets were increased as the sample size increased, the latter being guided by the Monte Carlo errors (White et al., 2011).

## 3 Results

Frequencies and percentages of alcohol use according to GAD and DTC motives, are presented in Table 1 with results from logistic models in Tables 2, 3 and 4.

**Table 1:** Frequencies and percentages for the main variables (available data).

		Frequent Drinking		Frequent Bingeing		Hazardous Drinking		Harmful Drinking	
		Age 18	Age 21	Age 18	Age 21	Age 18	Age 21	Age 18	Age 21
**Whole sample**		939 25.9%	845 40.9%	516 14.2%	706 32.6%	1551 42.8%	1246 57.6%	209 5.8%	280 12.9%

**GAD**	**No**	826 25.3%	786 40.3%	460 14.1%	635 32.6%	1382 42.3%	1118 57.3%	180 5.5%	247 12.7%
	**Yes**	62 32.1%	58 46.4%	36 18.7%	41 32.8%	99 51.3%	79 63.2%	20 10.4%	25 20.0%

**DTC**	**Low**	565 20.4%	658 39.0%	272 9.8%	516 30.6%	934 33.7%	907 53.8%	63 2.3%	172 10.2%
	**High**	373 44.3%	223 47.9%	241 28.6%	185 39.7%	614 72.8%	333 71.5%	146 17.3%	105 22.5%

**GAD** **(Low DTC stratum)**	**No**	520 20.3%	598 38.5%	257 10.0%	470 30.3%	866 33.8%	830 53.4%	59 2.3%	156 10.1%
	**Yes**	16 15.8%	29 42.7%	7 6.93%	19 27.9%	33 32.7%	38 55.9%	<5 <5%	10 14.7%

**GAD** **(High DTC stratum)**	**No**	305 43.5%	184 47.4%	201 28.7%	161 41.5%	514 73.3%	283 72.9%	121 17.3%	89 22.9%
	**Yes**	46 50.6%	29 51.8%	28 30.8%	21 37.5%	65 71.4%	40 71.4%	17 18.7%	14 25.0%

**Table 2:** Logistic regressions examining the associations of generalized anxiety disorder at age 18 with alcohol use at age 18 and 21.

		Age 18 Available data (n as shown)			Age 18 Imp#1 (n = 3625)		Age 21 Available data (n as shown)			Age 21 Imp#1 (n = 3625)	
	Model	N	OR [95% CI]	p-value	OR [95% CI]	p-value	N	OR [95% CI]	p-value	OR [95% CI]	p-value
**Frequent Drinking**	Model 1	3462	1.40 [1.02, 1.91]	.036	1.41 [1.03, 1.93]	.030	2076	1.28 [0.89, 1.84]	.178	1.26 [0.88, 1.80]	.204
	Model 2	2603	1.71 [1.19, 2.45]	.004	1.61 [1.17, 2.21]	.003	1611	1.34 [0.88, 2.06]	.176	1.38 [0.95, 2.00]	.091
	Model 3	1832	1.76 [1.13, 2.76]	.013	1.57 [1.13, 2.16]	.007	1213	1.77 [1.05, 3.00]	.033	1.38 [0.94, 2.03]	.097
	Model 4	1535	1.67 [0.99, 2.82]	.055	1.50 [1.07, 2.09]	.017	1043	1.44 [0.79, 2.63]	.232	1.34 [0.91, 1.99]	.138

**Frequent Bingeing**	Model 1	3462	1.40 [0.96, 2.04]	.079	1.39 [0.96, 2.02]	.083	2076	1.01 [0.69, 1.49]	.953	1.01 [0.69, 1.47]	.968
	Model 2	2603	1.66 [1.08, 2.57]	.021	1.54 [1.06, 2.26]	.025	1611	0.94 [0.60, 1.49]	.799	1.10 [0.75, 1.62]	.618
	Model 3	1832	1.81 [1.06, 3.09]	.031	1.51 [1.03, 2.22]	.034	1213	1.03 [0.60, 1.78]	.913	1.07 [0.72, 1.60]	.724
	Model 4	1535	1.67 [0.88, 3.18]	.120	1.45 [0.97, 2.15]	.068	1043	0.75 [0.40, 1.43]	.390	1.06 [0.71, 1.58]	.789

**Hazardous Drinking**	Model 1	3462	1.44 [1.08, 1.92]	.014	1.44 [1.08, 1.93]	.014	2076	1.28 [0.88, 1.86]	.197	1.23 [0.85, 1.79]	.279
	Model 2	2603	1.64 [1.17, 2.30]	.004	1.52 [1.13, 2.03]	.005	1611	1.31 [0.85, 2.01]	.226	1.30 [0.89, 1.90]	.174
	Model 3	1832	2.10 [1.37, 3.22]	.001	1.47 [1.09, 1.98]	.011	1213	2.16 [1.21, 3.84]	.009	1.29 [0.88, 1.89]	.200
	Model 4	1535	1.98 [1.21, 3.25]	.007	1.41 [1.03, 1.92]	.030	1043	1.86 [0.99, 3.49]	.054	1.26 [0.85, 1.87]	.256

**Harmful Drinking**	Model 1	3462	1.98 [1.22, 3.23]	.006	1.99 [1.22, 3.23]	.006	2076	1.72 [1.09, 2.73]	.020	1.67 [1.11, 2.51]	.014
	Model 2	2603	2.48 [1.42, 4.33]	.001	2.05 [1.25, 3.34]	.004	1611	1.51 [0.86, 2.67]	.152	1.79 [1.18, 2.71]	.006
	Model 3	1832	3.55 [1.90, 6.63]	<.001	1.97 [1.20, 3.25]	.008	1213	1.47 [0.75, 2.88]	.258	1.77 [1.16, 2.70]	.008
	Model 4	1535	4.10 [1.88, 8.93]	<.001	1.87 [1.12, 3.12]	.017	1043	1.29 [0.57, 2.91]	.536	1.68 [1.09, 2.60]	.020

Model 1 = unadjusted; model 2 = adjusted for sociodemographic confounders: gender, maternal education, family income, housing tenure, and social class; model 3 = additionally adjusted for parental confounders: parental depression, anxiety, alcohol use, and tobacco use; model 4 = additionally adjusted for adolescent confounders: tobacco use, cannabis use, drinking frequency, binge drinking, conduct problems, and emotional symptoms.

**Table 3:** Logistic regressions examining the associations of drinking to cope motives at age 18 with alcohol use at age 18 and 21.

		Age 18 Available data (n as shown)			Age 18 Imp#1 (n = 3625)		Age 21 Available data (n as shown)			Age 21 Imp#1 (n = 3625)	
	Model	N	OR [95% CI]	p-value	OR [95% CI]	p-value	N	OR [95% CI]	p-value	OR [95% CI]	p-value
**Frequent Drinking**	Model 1	3617	3.10 [2.63, 3.65]	<.001	3.10 [2.63, 3.65]	<.001	2152	1.43 [1.17, 1.76]	.001	1.43 [1.18, 1.74]	<.001
	Model 2	2730	3.15 [2.59, 3.82]	<.001	3.33 [2.82, 3.94]	<.001	1678	1.59 [1.24, 2.02]	<.001	1.50 [1.23, 1.84]	<.001
	Model 3	1915	2.84 [2.25, 3.59]	<.001	3.26 [2.75, 3.87]	<.001	1258	1.63 [1.22, 2.16]	.001	1.45 [1.18, 1.79]	<.001
	Model 4	1607	2.46 [1.88, 3.21]	<.001	3.00 [2.52, 3.57]	<.001	1084	1.50 [1.10, 2.06]	.012	1.37 [1.10, 1.69]	.005

**Frequent Bingeing**	Model 1	3617	3.68 [3.03, 4.47]	<.001	3.69 [3.03, 4.48]	<.001	2152	1.49 [1.21, 1.85]	<.001	1.51 [1.24, 1.84]	<.001
	Model 2	2730	3.65 [2.91, 4.60]	<.001	3.85 [3.16, 4.69]	<.001	1678	1.61 [1.26, 2.06]	<.001	1.58 [1.29, 1.93]	<.001
	Model 3	1915	3.34 [2.52, 4.43]	<.001	3.74 [3.06, 4.56]	<.001	1258	1.61 [1.21, 2.14]	.001	1.52 [1.23, 1.87]	<.001
	Model 4	1607	3.14 [2.27, 4.36]	<.001	3.44 [2.80, 4.23]	<.001	1084	1.48 [1.08, 2.03]	.015	1.45 [1.17, 1.80]	.001

**Hazardous Drinking**	Model 1	3617	5.28 [4.45, 6.27]	<.001	5.29 [4.46, 6.27]	<.001	2152	2.15 [1.72, 2.69]	<.001	2.19 [1.75, 2.74]	<.001
	Model 2	2730	4.81 [3.95, 5.86]	<.001	5.44 [4.58, 6.47]	<.001	1678	2.24 [1.73, 2.90]	<.001	2.28 [1.81, 2.86]	<.001
	Model 3	1915	4.81 [3.79, 6.10]	<.001	5.32 [4.47, 6.33]	<.001	1258	2.14 [1.58, 2.90]	<.001	2.21 [1.75, 2.79]	<.001
	Model 4	1607	4.34 [3.32, 5.68]	<.001	5.01 [4.19, 5.99]	<.001	1084	2.12 [1.52, 2.96]	<.001	2.12 [1.67, 2.69]	<.001

**Harmful Drinking**	Model 1	3617	9.01 [6.63, 12.25]	<.001	9.00 [6.62, 12.24]	<.001	2152	2.56 [1.96, 3.35]	<.001	2.73 [2.13, 3.51]	<.001
	Model 2	2730	8.62 [5.99, 12.41]	<.001	9.14 [6.71, 12.44]	<.001	1678	2.75 [2.02, 3.73]	<.001	2.83 [2.19, 3.65]	<.001
	Model 3	1915	8.02 [5.18, 12.42]	<.001	8.82 [6.45, 12.04]	<.001	1258	2.52 [1.76, 3.59]	<.001	2.70 [2.09, 3.50]	<.001
	Model 4	1607	7.06 [4.17, 11.96]	<.001	7.97 [5.81, 10.95]	<.001	1084	2.33 [1.56, 3.48]	<.001	2.46 [1.88, 3.22]	<.001

Model 1 = unadjusted; model 2 = adjusted for sociodemographic confounders: gender, maternal education, family income, housing tenure, and social class; model 3 = additionally adjusted for parental confounders: parental depression, anxiety, alcohol use, and tobacco use; model 4 = additionally adjusted for adolescent confounders: tobacco use, cannabis use, drinking frequency, binge drinking, conduct problems, and emotional symptoms.

**Table 4:** Logistic regressions examining the interactions between generalized anxiety disorder and drinking to cope motives at age 18 on alcohol use at age 18 and 21.

		Age 18 Available data (n as shown)			Age 18 Imp#1 (n = 3625)		Age 21 Available data (n as shown)			Age 21 Imp#1 (n = 3625)	
	Model	N	OR [95% CI]	p-value	OR [95% CI]	p-value	N	OR [95% CI]	p-value	OR [95% CI]	p-value
**Frequent Drinking**	*Stratum specific*										
	Low DTC	2660	0.74 [0.43, 1.27]	.270	0.76 [0.44, 1.30]	.315	1621	1.19 [0.73, 1.94]	.493	1.16 [0.71, 1.89]	.550
	High DTC	792	1.33 [0.86, 2.06]	.204	1.34 [0.87, 2.06]	.188	444	1.19 [0.68, 2.09]	.542	1.17 [0.68, 2.00]	.578
	Interaction	3452	1.80 [0.90, 3.62]	.098	1.77 [0.88, 3.54]	.108	2065	1.00 [0.48, 2.11]	.994	1.00 [0.49, 2.04]	.991

**Frequent Bingeing**	*Stratum specific*										
	Low DTC	2660	0.67 [0.31, 1.45]	.309	0.67 [0.31, 1.47]	.319	1621	0.89 [0.52, 1.53]	.683	0.89 [0.52, 1.50]	.651
	High DTC	792	1.11 [0.69, 1.78]	.678	1.15 [0.72, 1.84]	.557	444	0.85 [0.47, 1.51]	.570	0.91 [0.51, 1.61]	.736
	Interaction	3452	1.66 [0.67, 4.12]	.278	1.71 [0.69, 4.25]	.248	2065	0.95 [0.43, 2.09]	.892	1.02 [0.46, 2.27]	.955

**Hazardous Drinking**	*Stratum specific*										
	Low DTC	2660	0.95 [0.62, 1.45]	.810	0.96 [0.63, 1.47]	.850	1621	1.10 [0.68, 1.80]	.693	1.01 [0.64, 1.59]	.966
	High DTC	792	0.91 [0.56, 1.48]	.701	0.92 [0.57, 1.49]	.737	444	0.93 [0.50, 1.73]	.813	0.96 [0.53, 1.75]	.905
	Interaction	3452	0.96 [0.50, 1.82]	.896	0.96 [0.50, 1.82]	.899	2065	0.84 [0.38, 1.85]	.667	0.95 [0.45, 2.01]	.903

**Harmful Drinking**	*Stratum specific*										
	Low DTC	2660	1.30 [0.40, 4.21]	.664	1.30 [0.40, 4.23]	.659	1621	1.54 [0.77, 3.08]	.218	1.56 [0.78, 3.11]	.208
	High DTC	792	1.10 [0.63, 1.93]	.737	1.12 [0.64, 1.96]	.693	444	1.12 [0.58, 2.14]	.733	1.08 [0.59, 2.00]	.798
	Interaction	3452	0.85 [0.23, 3.13]	.805	0.86 [0.23, 3.17]	.820	2065	0.73 [0.28, 1.87]	.507	0.69 [0.28, 1.71]	.428

Unadjusted model. Stratified analysis: associations of generalized anxiety disorder at age 18 with alcohol use outcomes at age 18 and 21 in each stratum of drinking to cope motives. Interaction term: interaction of GAD x DTC at age 18 on alcohol use outcomes at age 18 and 21.

### 3.1 Associations between GAD and alcohol use

#### 3.1.1 Cross-sectional

At age 18, there was evidence of a positive association between GAD and all four alcohol outcomes. In unadjusted analyses with the available data, GAD was associated with more frequent drinking (OR 1.40, 95% CI 1.02–1.91, p = .036), hazardous drinking (OR 1.44, 95% CI 1.08–1.92, p = .014) and harmful drinking (OR 1.98, 95% CI 1.22–3.23, p = .006). There was only very weak evidence that GAD was associated with more frequent bingeing (OR 1.40, 95% CI 0.96–2.04, p = .079). For hazardous and harmful drinking, the associations were robust to adjustment for sociodemographic, parental, and adolescent confounders, whereas for frequent drinking and frequent bingeing associations were attenuated (Table 2). Following imputation, it was clear that sample reduction was driving the instability in estimates for these more problematic alcohol outcomes. Imputed results show confounders to have a more modest impact on associations between GAD and alcohol outcomes (Supplementary Table 3).

#### 3.1.2 Longitudinal

Table 2 shows the associations between adolescent GAD and alcohol use three years later were weaker than the cross-sectional associations. GAD increased the odds of harmful drinking at age 21 (available data unadjusted OR 1.72, 95% CI 1.09–2.73, p = .020), but there was no clear evidence of a longitudinal relationship between GAD and the other alcohol use outcomes. Imputed results showed little attenuation due to the range of confounders considered (fully adjusted imputation 1 OR 1.68, 95% CI 1.09–2.60, p = .020).

### 3.2 Associations between GAD and DTC

The odds of DTC were three times higher in individuals with GAD compared to those without GAD (available data unadjusted OR 3.23, 95% CI 2.41–4.34, p < .001). This association remained after adjusting for confounders (Supplementary Table 4).

### 3.3 Associations between DTC and alcohol use

DTC was strongly associated with all alcohol outcomes at both ages (Table 3). Like the associations between GAD and the alcohol outcomes, associations between DTC and alcohol use at age 18 increased from frequent drinking (available data unadjusted OR 3.10, 95% CI 2.63–3.65, p < .001) to harmful drinking (available data unadjusted OR 9.01, 95% CI 6.63–12.25, p < .001). Associations were robust to adjustment for confounders. This pattern was also evident at age 21, but point estimates were smaller.

### 3.4 Interactions between GAD and DTC on alcohol use

There was no clear evidence to support the hypothesis that associations between GAD and alcohol use outcomes would be stronger in people with high DTC motives (Table 4).

### 3.5 Attrition

Analyses with the available data revealed problem drinkers at age 18 were less likely to provide complete outcome data at age 21 (frequent drinkers OR 0.74, 95% CI 0.64–0.86, p < .001; frequent bingers OR 0.63, 95% CI 0.52–0.76, p < .001; hazardous drinkers OR 0.74, 95% CI 0.65–0.84, p < .001; harmful drinkers OR 0.60, 95% CI 0.46–0.80, p < .001). However, there was no clear evidence of an association between GAD at age 18 and completeness of outcome data at age 21 (OR 1.07, 95% CI 0.82–1.39, p = .62).

### 3.6 Sensitivity analyses

Results shown in Supplementary Tables 1 to 6 indicate our conclusions are consistent across the various imputed datasets. In addition, the inclusion of non-drinkers had little impact on the estimated association between GAD and alcohol use at either 18 or 21 years (Supplementary Table 7). Conclusions were also robust to the removal of other internalizing disorders from the GAD reference group (Supplementary Table 8).

## 4 Discussion

Consistent with self-medication theory, GAD at age 18 was positively associated with concurrent frequent drinking, frequent bingeing, hazardous drinking, and harmful drinking, in our sample of late adolescent drinkers. Although associations with hazardous and harmful drinking were robust to adjustment for confounders, associations with frequent drinking and frequent bingeing were attenuated. GAD at age 18 was prospectively associated with more harmful drinking at age 21, consistent with self-medication theory. However, these findings are contrary to some previous studies which have found no clear evidence of a longitudinal relationship between adolescent GAD and later problem drinking (Abram et al., 2015; Wolitzky-Taylor et al., 2012; Zimmermann et al., 2003). We found no clear evidence of a prospective relationship between GAD and frequent drinking, frequent bingeing, and hazardous drinking in early adulthood.

This same pattern has been observed with other anxiety disorders where anxiety is more strongly positively associated with alcohol problems/disorders than with alcohol consumption levels (Dyer et al., 2019; Schry and White, 2013). This suggests the self-medication hypothesis and tension-reducing drinking may be most pertinent for problem drinkers. Associations between anxiety and general consumption may be more context-dependent, which could explain the weaker associations. For example, there may be situational or individual difference variables which moderate the extent to which individuals with anxiety drink more or more frequently. Perhaps at the most severe forms of drinking, there may be common biological (Agoglia and Herman, 2018), cognitive (Chow et al., 2018) and/or environmental vulnerabilities (Jones et al., 2018) that increase the risk of both anxiety disorders and alcohol problems.

We also predicted associations between GAD and alcohol outcomes would be stronger in individuals who endorse high (vs. low) DTC motives. However, there was no clear evidence of an interaction between GAD and DTC. Our findings were consistent across the three imputed datasets.

The present study has several limitations. First, observational studies have inherent methodological limitations due to the absence of randomization, which precludes causal inferences from the data. Reverse causation is a possibility in our cross-sectional data. We adjusted for several potential confounders, but there may still be residual confounding. A Mendelian randomization study, using genetic variants associated with anxiety, would help to determine whether anxiety causes problem drinking by eliminating the impact of confounding and reverse causation (Chao et al., 2017; Lawlor et al., 2008). Second, self-report measures of alcohol consumption and motivations for drinking may be subject to recall or social desirability biases and thus measurement error. Third, a lack of clear evidence for prospective associations between GAD and frequent drinking and frequent bingeing may be due to the use of single-item measures for these outcomes. Converting these ordinal items to binary variables may have also resulted in reduced power. However, our results are consistent with other prospective cohort studies (Dyer et al., 2019), which suggests these measures are valid. Fourth, there was evidence of differential attrition at follow up; problem drinkers at age 18 were more likely to have missing outcome data at age 21. A smaller sample of problem drinkers at age 21 may have biased our results with the available data towards the null. However, when we included auxiliary data in multiple imputation models there was stronger evidence of an association between GAD and harmful drinking. By using multiple imputation, we increased the likelihood that a Missing at random assumption could be made, therefore reducing the likelihood of bias. Finally, as the UK has one of the highest alcohol consumption levels for adolescents in Europe (Hibell et al., 2012), the findings may not be generalizable to other countries. Despite these limitations, to the best of our knowledge this is the largest study to investigate prospective associations between GAD in adolescence and alcohol use in early adulthood with a series of multiply-imputed datasets to examine the robustness of the available-data estimates, and statistical adjustment for a range of important confounders.

The relationship between GAD and alcohol use may be qualitatively different in adolescence compared to emerging adulthood, as a result of biological or social context changes over time. Adolescence is a developmental period characterized by greater propensity for risk-taking, impulsivity (Arnett, 1992), sensation seeking and susceptibility to peer influences (Albert and Steinberg, 2011). Behavioral and neuroimaging research has also shown adolescents have increased reward sensitivity and reduced cognitive control than adults (Albert and Steinberg, 2011). In addition, as the legal age for purchasing alcohol in the UK is 18, drinking at age 18 might be considered novel and exciting. Late adolescence may therefore be a vulnerable period where the relationship between anxiety and alcohol use is more pronounced. A replication study in a USA cohort, at comparable time points related to the legal minimum drinking age, (i.e., age 21 vs. 24) would also test the changing social context interpretation. We could also examine the importance of age by repeating analyses in an older sample and using an outcome measure that captures longitudinal change in alcohol use.

Changes in the relationship between GAD and alcohol use from age 18 to 21 could be explained by changes in alcohol expectancies - beliefs about the positive or negative behavioral, emotional and cognitive effects of alcohol (Baer, 2002). Individuals who have higher (vs. lower) expectancies for alcohol to be anxiety reducing, have a stronger correlation between anxiety and alcohol use (Kushner et al., 1994) and are more likely to endorse a self-medicating style of drinking (Kushner et al., 2000). GAD may initially lead to increased alcohol consumption to self-medicate anxiety symptoms. After several years, alcohol may exacerbate anxiety symptoms, which could result in a reduction of drinking. Anxious individuals may also replace alcohol with prescription medication or psychological therapies to manage their symptoms. Future research examining changes in alcohol expectancies and treatments over time would be informative.

There are several possible explanations why DTC did not moderate the relationship between GAD and alcohol use. First, differences between high and low DTC individuals may have been undetected because of inadequate statistical power, a common criticism of interaction tests (Marshall, 2007). Second, ongoing work from our research group suggests DTC may be more relevant to short term state anxiety, than chronic anxiety such as GAD. Third, self-medicated drinking may be more greatly endorsed by adults than adolescents (Hussong et al., 2011). Fourth, moderation effects of DTC may be masked in an adolescent sample as young people are motivated to drink for a variety of reasons (Kuntsche et al., 2005). There may be meaningful differences between individuals who drink to cope only, and those who drink to cope *and* drink for social, conformity, and/or enhancement motives. Excluding the latter individuals from our DTC variable may have altered the results (misclassification or measurement error). Fifth, global/dispositional measures of DTC may not be sensitive enough as they fail to account for within-person variation in drinking motives (O’Hara et al., 2014). People who drink to cope also cope in other ways (Todd et al., 2004), and self-medication with alcohol may depend on situational variables (Arbeau et al., 2011). Finally, DTC motives may only occur in a subgroup of individuals with anxiety (Kushner et al., 2000). Possible factors affecting choice of alcohol as a method of coping include availability, modelling of parents’ drinking behavior, culture/religion, socioeconomic status, biological predisposition, and alcohol expectancies. Follow up research examining how and why the relationship between GAD and alcohol use changes over time, reconsidering the role of DTC motives, is required.

## 5 Conclusions

There is considerable public health interest in identifying adolescent antecedents of drinking patterns and problems in adulthood. Although GAD in adolescence predicted concurrent frequent drinking, frequent bingeing, hazardous drinking and harmful drinking, associations remained for only harmful drinking in early adulthood. There was no clear evidence of an interaction between GAD and DTC on alcohol use in adolescence or early adulthood. Additional epidemiological and experimental approaches are required to further examine the roles of anxiety and DTC in the aetiology of alcohol problems, in order to inform tailored prevention and intervention strategies.

## Role of funding source

This research was funded by the University of Bristol MRC Addiction Research Clinical Training program (MARC), and the MRC Integrative Epidemiology Unit (IEU) at the University of Bristol. MRM is a program lead in the MRC IEU (MC_UU_00011/7). MD and MRM are members of the UK Centre for Tobacco and Alcohol Studies. We acknowledge funding from the MRC and Alcohol Research UK (MR/L022206/1) which supports JH. The work was undertaken with the support of The Centre for the Development and Evaluation of Complex Interventions for Public Health Improvement (DECIPHer), a UKCRC Public Health Research Centre of Excellence. Joint funding (MR/KO232331/1) from the British Heart Foundation, Cancer Research UK, Economic and Social Research Council, Medical Research Council, the Welsh Government and the Wellcome Trust, under the auspices of the UK Clinical Research Collaboration, is gratefully acknowledged. We also acknowledge funding from the NIHR School of Public Health Research, NIHR Health Protection Research Unit in Evaluation, and NIHR BRC at Bristol.

## Contributors

All authors contributed to the study design and analysis plan. MD analyzed the available data. JH produced and analyzed the multiply-imputed datasets. MD wrote the manuscript with input from all authors. All authors have approved the final article.

## Declaration of Competing Interest

No conflict declared.
