# Supplementary material for: Alcohol use in late adolescence and early adulthood: The role of generalized anxiety disorder and drinking to cope motives
Source: Drug Alcohol Depend. 2019 Nov 1;204:107480. doi: 10.1016/j.drugalcdep.2019.04.044 (PMC6891250; doi:10.1016/j.drugalcdep.2019.04.044)
Supplement: Supplementary file 1 [file mmc1.docx]

**Supplementary Material for the Article:**

Alcohol use in late adolescence and early adulthood:

The role of generalized anxiety disorder and drinking to cope motives

**This material supplements, but does not replace, the peer-reviewed paper in**

**Drug and Alcohol Dependence.**

Maddy L. Dyer^a, b, c^, maddy.dyer@bristol.ac.uk

Jon Heron^b, c^, jon.heron@bristol.ac.uk

Matthew Hickman^b,c^, matthew.hickman@bristol.ac.uk

Marcus R. Munafò^a, c^, marcus.munafo@bristol.ac.uk

1. School of Psychological Science, University of Bristol, Bristol, United Kingdom
2. Population Health Sciences, Bristol Medical School, University of Bristol, Bristol, United Kingdom
3. MRC Integrative Epidemiology Unit (IEU), University of Bristol, Bristol, United Kingdom

**Correspondence:**

Maddy Dyer

School of Psychological Science, University of Bristol

12a Priory Road, Bristol, BS8 1TU, United Kingdom

E-mail: maddy.dyer@bristol.ac.uk.

**Supplementary Figure 1. Timeline of study variables.**


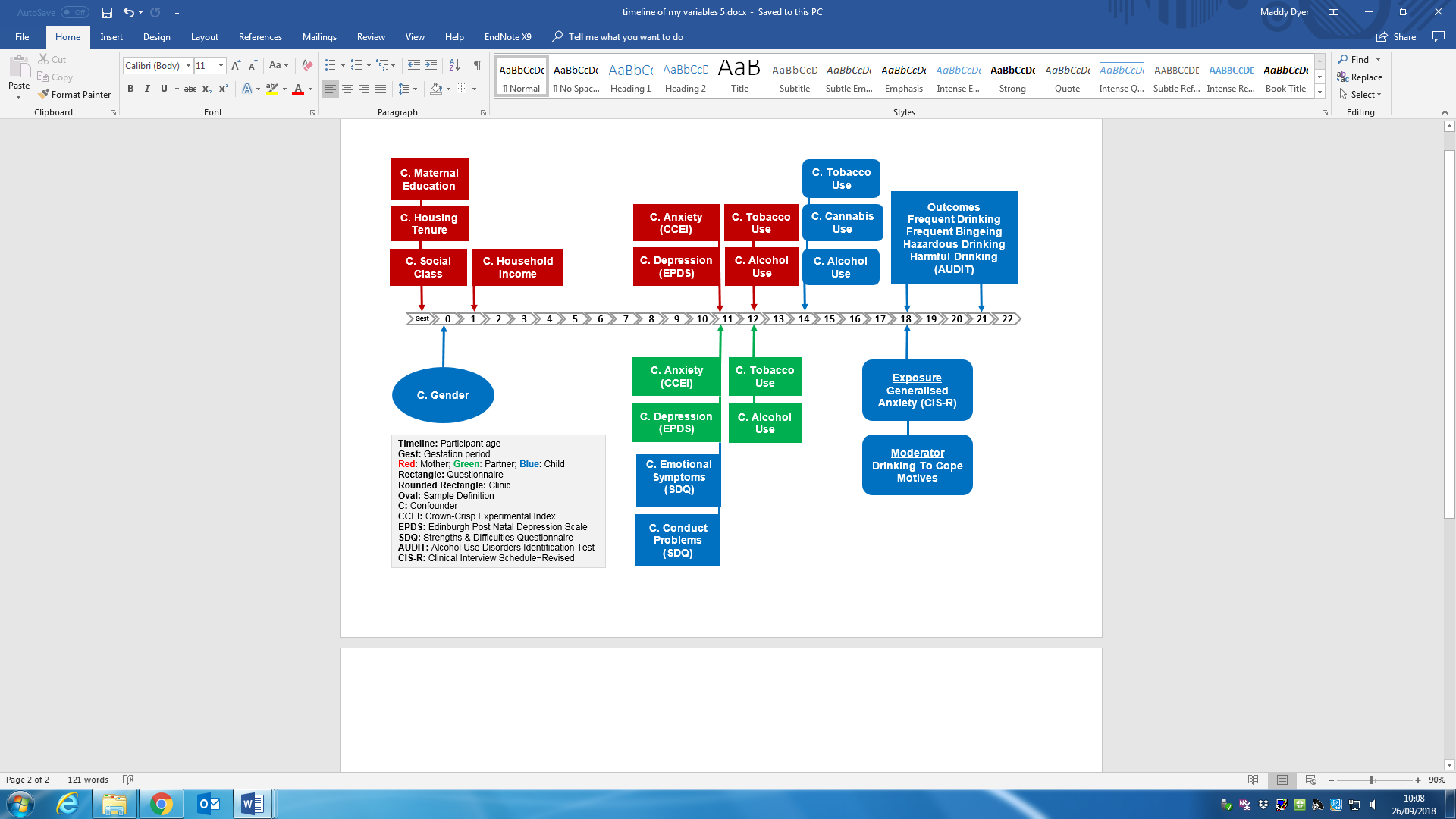


**Supplementary Table 1. Frequencies and percentages for the main variables (cross-sectional).**

|  |  | **Frequent Drinking (18)** | | | | **Frequent Bingeing (18)** | | | | **Hazardous Drinking (18)** | | | | **Harmful Drinking (18)** | | | |
| --- | --- | --- | --- | --- | --- | --- | --- | --- | --- | --- | --- | --- | --- | --- | --- | --- | --- |
|  |  | **AD** | **Imp#1** | **Imp#2** | **Imp#3** | **AD** | **Imp#1** | **Imp#2** | **Imp#3** | **AD** | **Imp#1** | **Imp#2** | **Imp#3** | **AD** | **Imp#1** | **Imp#2** | **Imp#3** |
| **Whole sample** |  | 939  25.9% | 25.9% (0.7%) | 25.9% (0.7%) | 25.4% (0.7%) | 516  14.2% | 14.2% (0.6%) | 14.6% (0.6%) | 14.9% (0.6%) | 1551  42.8% | 42.8% (0.8%) | 42.8% (0.8%) | 43.0% (0.8%) | 209  5.8% | 5.8% (0.4%) | 5.9% (0.4%) | 6.2% (0.4%) |
| **GAD** | **No** | 826  25.3% | 25.5% (0.7%) | 25.6% (0.7%) | 25.0% (0.7%) | 460  14.1% | 14.0% (0.6%) | 14.4% (0.6%) | 14.7% (0.6%) | 1382  42.3% | 42.3% (0.8%) | 42.4% (0.8%) | 42.4% (0.8%) | 180  5.5% | 5.5% (0.4%) | 5.7% (0.4%) | 5.9% (0.4%) |
|  | **Yes** | 62  32.1% | 32.6% (3.4%) | 31.6% (3.3%) | 31.6% (3.2%) | 36  18.7% | 18.5% (2.8%) | 17.9% (2.6%) | 18.9% (2.7%) | 99  51.3% | 51.4% (3.6%) | 50.3% (3.5%) | 51.0% (3.6%) | 20  10.4% | 10.4% (2.2%) | 10.1% (2.1%) | 10.6% (2.1%) |
|  |  |  |  |  |  |  |  |  |  |  |  |  |  |  |  |  |  |
| **DTC** | **Low** | 565  20.4% | 20.4% (0.8%) | 20.3% (0.7%) | 19.6% (0.7%) | 272  9.8% | 9.8% (0.6%) | 10.1% (0.6%) | 10.2% (0.6%) | 934  33.7% | 33.6% (0.9%) | 33.9% (0.9%) | 34.0% (0.9%) | 63  2.3% | 2.3% (0.3%) | 2.4% (0.3%) | 2.6% (0.3%) |
|  | **High** | 373  44.3% | 44.2% (1.7%) | 44.1% (1.7%) | 43.4% (1.6%) | 241  28.6% | 28.7% (1.6%) | 29.2% (1.5%) | 29.7% (1.5%) | 614  72.8% | 72.8% (1.5%) | 71.9% (1.6%) | 71.1% (1.5%) | 146  17.3% | 17.3% (1.3%) | 17.2% (1.3%) | 17.2% (1.3%) |
|  |  |  |  |  |  |  |  |  |  |  |  |  |  |  |  |  |  |
| **GAD**  **(Low DTC stratum)** | **No** | 520  20.3% | 20.5% (0.8%) | 20.5% (0.8%) | 19.7% (0.8%) | 257  10.0% | 9.9% (0.6%) | 10.3% (0.6%) | 10.3% (0.6%) | 866  33.8% | 33.7% (0.9%) | 33.9% (0.9%) | 34.0% (0.9%) | 59  2.3% | 2.2% (0.3%) | 2.4% (0.3%) | 2.6% (0.3%) |
|  | **Yes** | 16  15.8% | 16.4% (3.7%) | 15.1% (3.5%) | 16.5% (3.6%) | 7  6.93% | 6.9% (2.5%) | 6.7% (2.4%) | 6.9% (2.5%) | 33  32.7% | 32.8% (4.7%) | 32.5% (4.6%) | 33.8% (4.6%) | <5  <5% | 2.9% (1.7%) | 3.2% (1.8%) | 3.8% (2.0%) |
|  |  |  |  |  |  |  |  |  |  |  |  |  |  |  |  |  |  |
| **GAD**  **(High DTC stratum)** | **No** | 305  43.5% | 43.3% (1.8%) | 43.3% (1.8%) | 42.9% (1.8%) | 201  28.7% | 28.4% (1.7%) | 29.0% (1.7%) | 29.6% (1.7%) | 514  73.3% | 73.0% (1.6%) | 72.1% (1.6%) | 71.1% (1.7%) | 121  17.3% | 17.1% (1.4%) | 16.9% (1.4%) | 17.0% (1.4%) |
|  | **Yes** | 46  50.6% | 50.6% (5.2%) | 49.5% (5.0%) | 49.1% (5.3%) | 28  30.8% | 31.3% (4.8%) | 31.0% (4.8%) | 32.2% (4.7%) | 65  71.4% | 71.4% (4.7%) | 69.5% (4.9%) | 68.9% (4.7%) | 17  18.7% | 18.8% (4.1%) | 18.2% (3.9%) | 18.7% (4.0%) |

Numbers in the brackets indicate the precision around the estimated percentage for the imputed data.

AD = available data; Imp#1: n = 3625; 100 imputations; Imp#2: n = 4600; 100 imputations; Imp#3: n = 9278; 200 imputations.

**Supplementary Table 2. Frequencies and percentages for the main variables (longitudinal)**

|  |  | **Frequent Drinking (21)** | | | | **Frequent Bingeing (21)** | | | | **Hazardous Drinking (21)** | | | | **Harmful Drinking (21)** | | | |
| --- | --- | --- | --- | --- | --- | --- | --- | --- | --- | --- | --- | --- | --- | --- | --- | --- | --- |
|  |  | **AD** | **Imp#1** | **Imp#2** | **Imp#3** | **AD** | **Imp#1** | **Imp#2** | **Imp#3** | **AD** | **Imp#1** | **Imp#2** | **Imp#3** | **AD** | **Imp#1** | **Imp#2** | **Imp#3** |
| **Whole sample** |  | 845  40.9% | 40.5% (1.0%) | 39.3% (1.0%) | 36.6% (0.8%) | 706  32.6% | 32.7% (1.0%) | 31.8% (0.9%) | 29.6% (0.8%) | 1246  57.6% | 58.4% (1.1%) | 57.7% (0.9%) | 55.6% (0.8%) | 280  12.9% | 13.7% (0.7%) | 13.7% (0.7%) | 13.2% (0.6%) |
| **GAD** | **No** | 786  40.3% | 40.2% (1.0%) | 38.9% (1.0%) | 36.2% (0.8%) | 635  32.6% | 32.6% (1.0%) | 31.9% (0.9%) | 29.6% (0.9%) | 1118  57.3% | 58.1% (1.1%) | 57.5% (1.0%) | 55.3% (0.9%) | 247  12.7% | 13.3% (0.7%) | 13.2% (0.7%) | 12.7% (0.6%) |
|  | **Yes** | 58  46.4% | 45.9% (4.3%) | 45.2% (4.0%) | 42.9% (3.8%) | 41  32.8% | 32.9% (4.1%) | 31.0% (3.6%) | 29.1% (3.5%) | 79  63.2% | 63.1% (4.3%) | 60.2% (3.9%) | 59.2% (4.1%) | 25  20.0% | 20.5% (3.2%) | 20.7% (3.2%) | 20.7% (3.1%) |
|  |  |  |  |  |  |  |  |  |  |  |  |  |  |  |  |  |  |
| **DTC** | **Low** | 658  39.0% | 38.5% (1.1%) | 37.2% (1.1%) | 34.8% (0.9%) | 516  30.6% | 30.5% (1.1%) | 29.7% (1.0%) | 27.5% (0.9%) | 907  53.8% | 54.2% (1.2%) | 53.5% (1.1%) | 51.0% (1.0%) | 172  10.2% | 10.5% (0.7%) | 10.4% (0.7%) | 10.1% (0.7%) |
|  | **High** | 223  47.9% | 47.2% (2.1%) | 45.9% (2.1%) | 42.2% (2.0%) | 185  39.7% | 39.8% (2.0%) | 38.7% (2.1%) | 36.0% (2.0%) | 333  71.5% | 72.2% (2.1%) | 71.3% (1.9%) | 69.9% (1.9%) | 105  22.5% | 24.3% (1.9%) | 24.2% (1.9%) | 22.8% (1.5%) |
|  |  |  |  |  |  |  |  |  |  |  |  |  |  |  |  |  |  |
| **GAD**  **(Low DTC stratum)** | **No** | 598  38.5% | 38.5% (1.2%) | 37.2% (1.1%) | 34.7% (1.0%) | 470  30.3% | 30.6% (1.1%) | 29.9% (1.0%) | 27.8% (1.0%) | 830  53.4% | 54.2% (1.2%) | 53.5% (1.1%) | 51.1% (1.1%) | 156  10.1% | 10.4% (0.7%) | 10.2% (0.7%) | 9.9% (0.6%) |
|  | **Yes** | 29  42.7% | 42.1% (5.9%) | 41.1% (5.4%) | 38.2% (5.1%) | 19  27.9% | 28.2% (5.3%) | 27.0% (4.8%) | 25.4% (4.7%) | 38  55.9% | 54.4% (5.6%) | 51.5% (5.5%) | 50.1% (5.6%) | 10  14.7% | 15.5% (4.5%) | 15.8% (4.1%) | 16.3% (4.3%) |
|  |  |  |  |  |  |  |  |  |  |  |  |  |  |  |  |  |  |
| **GAD**  **(High DTC stratum)** | **No** | 184  47.4% | 46.6% (2.3%) | 45.0% (2.3%) | 41.6% (2.2%) | 161  41.5% | 40.0% (2.3%) | 38.9% (2.3%) | 35.9% (2.1%) | 283  72.9% | 72.2% (2.1%) | 71.4% (2.1%) | 70.1% (2.1%) | 89  22.9% | 24.1% (2.1%) | 24.0% (1.9%) | 22.8% (1.8%) |
|  | **Yes** | 29  51.8% | 50.4% (6.4%) | 50.1% (6.2%) | 48.0% (6.0%) | 21  37.5% | 37.7% (6.3%) | 36.0% (5.7%) | 33.8% (5.8%) | 40  71.4% | 71.4% (5.6%) | 69.0% (6.0%) | 68.2% (5.6%) | 14  25.0% | 25.7% (5.3%) | 25.7% (5.1%) | 24.6% (5.1%) |

Numbers in the brackets indicate the precision around the estimated percentage for the imputed data.

AD = available data; Imp#1: n = 3625; 100 imputations; Imp#2: n = 4600; 100 imputations; Imp#3: n = 9278; 200 imputations.

**Supplementary Table 3. Logistic regressions examining the associations of generalized anxiety disorder at age 18 with alcohol use at age 18 and 21.**

|  |  | **Available data** | | | **Imp#1** | | **Imp#2** | | **Imp#3** | |
| --- | --- | --- | --- | --- | --- | --- | --- | --- | --- | --- |
|  | **Model** | **N** | **OR [95% CI]** | **p-value** | **OR [95% CI]** | **p-value** | **OR [95% CI]** | **p-value** | **OR [95% CI]** | **p-value** |
| **Age 18** |  |  |  |  |  |  |  |  |  |  |
| **Frequent Drinking** | Model 1 | 3462 | 1.40 [1.02, 1.91] | .036 | 1.41 [1.03, 1.93] | .030 | 1.34 [0.98, 1.84] | .068 | 1.38 [1.02, 1.85] | .037 |
|  | Model 2 | 2603 | 1.71 [1.19, 2.45] | .004 | 1.61 [1.17, 2.21] | .003 | 1.45 [1.06, 2.00] | .021 | 1.43 [1.05, 1.93] | .021 |
|  | Model 3 | 1832 | 1.76 [1.13, 2.76] | .013 | 1.57 [1.13, 2.16] | .007 | 1.42 [1.03, 1.96] | .034 | 1.38 [1.01, 1.88] | .041 |
|  | Model 4 | 1535 | 1.67 [0.99, 2.82] | .055 | 1.50 [1.07, 2.09] | .017 | 1.38 [0.99, 1.92] | .059 | 1.33 [0.97, 1.83] | .072 |
|  |  |  |  |  |  |  |  |  |  |  |
| **Frequent Bingeing** | Model 1 | 3462 | 1.40 [0.96, 2.04] | .079 | 1.39 [0.96, 2.02] | .083 | 1.29 [0.90, 1.85] | .165 | 1.29 [0.90, 1.86] | .165 |
|  | Model 2 | 2603 | 1.66 [1.08, 2.57] | .021 | 1.54 [1.06, 2.26] | .025 | 1.37 [0.95, 1.98] | .092 | 1.33 [0.92, 1.92] | .129 |
|  | Model 3 | 1832 | 1.81 [1.06, 3.09] | .031 | 1.51 [1.03, 2.22] | .034 | 1.34 [0.93, 1.95] | .118 | 1.29 [0.89, 1.86] | .173 |
|  | Model 4 | 1535 | 1.67 [0.88, 3.18] | .120 | 1.45 [0.97, 2.15] | .068 | 1.30 [0.89, 1.92] | .179 | 1.26 [0.86, 1.84] | .244 |
|  |  |  |  |  |  |  |  |  |  |  |
| **Hazardous Drinking** | Model 1 | 3462 | 1.44 [1.08, 1.92] | .014 | 1.44 [1.08, 1.93] | .014 | 1.37 [1.04, 1.82] | .026 | 1.41 [1.06, 1.88] | .020 |
|  | Model 2 | 2603 | 1.64 [1.17, 2.30] | .004 | 1.52 [1.13, 2.03] | .005 | 1.42 [1.07, 1.89] | .015 | 1.44 [1.08, 1.92] | .013 |
|  | Model 3 | 1832 | 2.10 [1.37, 3.22] | .001 | 1.47 [1.09, 1.98] | .011 | 1.37 [1.03, 1.82] | .030 | 1.37 [1.02, 1.84] | .034 |
|  | Model 4 | 1535 | 1.98 [1.21, 3.25] | .007 | 1.41 [1.03, 1.92] | .030 | 1.33 [0.99, 1.78] | .062 | 1.33 [0.98, 1.81] | .065 |
|  |  |  |  |  |  |  |  |  |  |  |
| **Harmful Drinking** | Model 1 | 3462 | 1.98 [1.22, 3.23] | .006 | 1.99 [1.22, 3.23] | .006 | 1.87 [1.15, 3.04] | .012 | 1.87 [1.16, 3.02] | .010 |
|  | Model 2 | 2603 | 2.48 [1.42, 4.33] | .001 | 2.05 [1.25, 3.34] | .004 | 1.89 [1.16, 3.09] | .011 | 1.89 [1.17, 3.06] | .009 |
|  | Model 3 | 1832 | 3.55 [1.90, 6.63] | <.001 | 1.97 [1.20, 3.25] | .008 | 1.81 [1.10, 3.00] | .020 | 1.81 [1.12, 2.93] | .015 |
|  | Model 4 | 1535 | 4.10 [1.88, 8.93] | <.001 | 1.87 [1.12, 3.12] | .017 | 1.74 [1.03, 2.92] | .037 | 1.73 [1.05, 2.84] | .032 |

Model 1 = unadjusted; model 2 = adjusted for sociodemographic confounders: gender, maternal education, family income, housing tenure, and social class; model 3 = additionally adjusted for parental confounders: parental depression, anxiety, alcohol use, and tobacco use; model 4 = additionally adjusted for adolescent confounders: tobacco use, cannabis use, drinking frequency, binge drinking, conduct problems, and emotional symptoms.

AD = available data; Imp#1: n = 3625; 100 imputations; Imp#2: n = 4600; 100 imputations; Imp#3: n = 9278; 200 imputations.

**Supplementary Table 3 (cont.).**

|  |  | **Available data** | | | **Imp#1** | | **Imp#2** | | **Imp#3** | |
| --- | --- | --- | --- | --- | --- | --- | --- | --- | --- | --- |
|  | **Model** | **N** | **OR [95% CI]** | **p-value** | **OR [95% CI]** | **p-value** | **OR [95% CI]** | **p-value** | **OR [95% CI]** | **p-value** |
| **Age 21** |  |  |  |  |  |  |  |  |  |  |
| **Frequent Drinking** | Model 1 | 2076 | 1.28 [0.89, 1.84] | .178 | 1.26 [0.88, 1.80] | .204 | 1.30 [0.93, 1.80] | .120 | 1.32 [0.93, 1.87] | .116 |
|  | Model 2 | 1611 | 1.34 [0.88, 2.06] | .176 | 1.38 [0.95, 2.00] | .091 | 1.40 [1.00, 1.97] | .052 | 1.41 [0.98, 2.02] | .063 |
|  | Model 3 | 1213 | 1.77 [1.05, 3.00] | .033 | 1.38 [0.94, 2.03] | .097 | 1.41 [1.00, 2.00] | .051 | 1.41 [0.98, 2.04] | .066 |
|  | Model 4 | 1043 | 1.44 [0.79, 2.63] | .232 | 1.34 [0.91, 1.99] | .138 | 1.40 [0.99, 2.00] | .060 | 1.40 [0.96, 2.04] | .079 |
|  |  |  |  |  |  |  |  |  |  |  |
| **Frequent Bingeing** | Model 1 | 2076 | 1.01 [0.69, 1.49] | .953 | 1.01 [0.69, 1.47] | .968 | 0.96 [0.68, 1.35] | .817 | 0.98 [0.68, 1.42] | .929 |
|  | Model 2 | 1611 | 0.94 [0.60, 1.49] | .799 | 1.10 [0.75, 1.62] | .618 | 1.03 [0.72, 1.46] | .880 | 1.03 [0.71, 1.50] | .871 |
|  | Model 3 | 1213 | 1.03 [0.60, 1.78] | .913 | 1.07 [0.72, 1.60] | .724 | 1.02 [0.71, 1.46] | .915 | 1.01 [0.69, 1.48] | .939 |
|  | Model 4 | 1043 | 0.75 [0.40, 1.43] | .390 | 1.06 [0.71, 1.58] | .789 | 1.01 [0.70, 1.46] | .941 | 1.02 [0.69, 1.50] | .919 |
|  |  |  |  |  |  |  |  |  |  |  |
| **Hazardous Drinking** | Model 1 | 2076 | 1.28 [0.88, 1.86] | .197 | 1.23 [0.85, 1.79] | .279 | 1.12 [0.80, 1.57] | .501 | 1.19 [0.84, 1.67] | .327 |
|  | Model 2 | 1611 | 1.31 [0.85, 2.01] | .226 | 1.30 [0.89, 1.90] | .174 | 1.17 [0.83, 1.64] | .364 | 1.23 [0.87, 1.75] | .232 |
|  | Model 3 | 1213 | 2.16 [1.21, 3.84] | .009 | 1.29 [0.88, 1.89] | .200 | 1.15 [0.82, 1.62] | .411 | 1.20 [0.84, 1.71] | .307 |
|  | Model 4 | 1043 | 1.86 [0.99, 3.49] | .054 | 1.26 [0.85, 1.87] | .256 | 1.14 [0.80, 1.62] | .462 | 1.20 [0.84, 1.72] | .325 |
|  |  |  |  |  |  |  |  |  |  |  |
| **Harmful Drinking** | Model 1 | 2076 | 1.72 [1.09, 2.73] | .020 | 1.67 [1.11, 2.51] | .014 | 1.70 [1.14, 2.54] | .010 | 1.70 [1.12, 2.58] | .013 |
|  | Model 2 | 1611 | 1.51 [0.86, 2.67] | .152 | 1.79 [1.18, 2.71] | .006 | 1.79 [1.19, 2.70] | .005 | 1.76 [1.16, 2.68] | .008 |
|  | Model 3 | 1213 | 1.47 [0.75, 2.88] | .258 | 1.77 [1.16, 2.70] | .008 | 1.77 [1.16, 2.69] | .008 | 1.72 [1.12, 2.65] | .013 |
|  | Model 4 | 1043 | 1.29 [0.57, 2.91] | .536 | 1.68 [1.09, 2.60] | .020 | 1.72 [1.11, 2.65] | .015 | 1.69 [1.08, 2.64] | .022 |

Model 1 = unadjusted; model 2 = adjusted for sociodemographic confounders: gender, maternal education, family income, housing tenure, and social class; model 3 = additionally adjusted for parental confounders: parental depression, anxiety, alcohol use, and tobacco use; model 4 = additionally adjusted for adolescent confounders: tobacco use, cannabis use, drinking frequency, binge drinking, conduct problems, and emotional symptoms.

AD = available data; Imp#1: n = 3625; 100 imputations; Imp#2: n = 4600; 100 imputations; Imp#3: n = 9278; 200 imputations.

**Supplementary Table 4. Logistic regressions examining the associations of generalized anxiety disorder at age 18 (exposure) with drinking to cope motives (outcome) at age 18.**

|  | **Available data** | | | **Imp#1** | | **Imp#2** | | **Imp#3** | |
| --- | --- | --- | --- | --- | --- | --- | --- | --- | --- |
| **Model** | **N** | **OR [95% CI]** | **p-value** | **OR [95% CI]** | **p-value** | **OR [95% CI]** | **p-value** | **OR [95% CI]** | **p-value** |
| Model 1 | 3477 | 3.23 [2.41, 4.34] | <.001 | 3.30 [2.46, 4.44] | <.001 | 3.18 [2.36, 4.28] | <.001 | 3.15 [2.35, 4.22] | <.001 |
| Model 2 | 2610 | 3.21 [2.28, 4.52] | <.001 | 3.16 [2.34, 4.25] | <.001 | 3.08 [2.28, 4.16] | <.001 | 3.05 [2.28, 4.09] | <.001 |
| Model 3 | 1833 | 3.48 [2.28, 5.32] | <.001 | 3.09 [2.28, 4.18] | <.001 | 2.98 [2.19, 4.05] | <.001 | 2.92 [2.17, 3.93] | <.001 |
| Model 4 | 1536 | 3.07 [1.88, 5.01] | <.001 | 3.01 [2.21, 4.09] | <.001 | 2.93 [2.14, 3.99] | <.001 | 2.86 [2.10, 3.88] | <.001 |

Model 1 = unadjusted; model 2 = adjusted for sociodemographic confounders: gender, maternal education, family income, housing tenure, and social class; model 3 = additionally adjusted for parental confounders: parental depression, anxiety, alcohol use, and tobacco use; model 4 = additionally adjusted for adolescent confounders: tobacco use, cannabis use, drinking frequency, binge drinking, conduct problems, and emotional symptoms.

AD = available data; Imp#1: n = 3625; 100 imputations; Imp#2: n = 4600; 100 imputations; Imp#3: n = 9278; 200 imputations.

**Supplementary Table 5. Logistic regressions examining the associations of drinking to cope motives at age 18 with alcohol use at age 18 and 21.**

|  |  | **Available data** | | | **Imp#1** | | **Imp#2** | | **Imp#3** | |
| --- | --- | --- | --- | --- | --- | --- | --- | --- | --- | --- |
|  | **Model** | **N** | **OR [95% CI]** | **p-value** | **OR [95% CI]** | **p-value** | **OR [95% CI]** | **p-value** | **OR [95% CI]** | **p-value** |
| **Age 18** |  |  |  |  |  |  |  |  |  |  |
| **Frequent Drinking** | Model 1 | 3617 | 3.10 [2.63, 3.65] | <.001 | 3.10 [2.63, 3.65] | <.001 | 3.10 [2.63, 3.65] | <.001 | 3.15 [2.70, 3.67] | <.001 |
|  | Model 2 | 2730 | 3.15 [2.59, 3.82] | <.001 | 3.33 [2.82, 3.94] | <.001 | 3.27 [2.77, 3.87] | <.001 | 3.28 [2.80, 3.84] | <.001 |
|  | Model 3 | 1915 | 2.84 [2.25, 3.59] | <.001 | 3.26 [2.75, 3.87] | <.001 | 3.21 [2.71, 3.80] | <.001 | 3.21 [2.73, 3.77] | <.001 |
|  | Model 4 | 1607 | 2.46 [1.88, 3.21] | <.001 | 3.00 [2.52, 3.57] | <.001 | 2.95 [2.48, 3.51] | <.001 | 2.97 [2.51, 3.51] | <.001 |
|  |  |  |  |  |  |  |  |  |  |  |
| **Frequent Bingeing** | Model 1 | 3617 | 3.68 [3.03, 4.47] | <.001 | 3.69 [3.03, 4.48] | <.001 | 3.66 [3.01, 4.44] | <.001 | 3.74 [3.08, 4.53] | <.001 |
|  | Model 2 | 2730 | 3.65 [2.91, 4.60] | <.001 | 3.85 [3.16, 4.69] | <.001 | 3.75 [3.08, 4.56] | <.001 | 3.78 [3.11, 4.60] | <.001 |
|  | Model 3 | 1915 | 3.34 [2.52, 4.43] | <.001 | 3.74 [3.06, 4.56] | <.001 | 3.65 [2.99, 4.45] | <.001 | 3.68 [3.02, 4.50] | <.001 |
|  | Model 4 | 1607 | 3.14 [2.27, 4.36] | <.001 | 3.44 [2.80, 4.23] | <.001 | 3.34 [2.72, 4.09] | <.001 | 3.38 [2.75, 4.16] | <.001 |
|  |  |  |  |  |  |  |  |  |  |  |
| **Hazardous Drinking** | Model 1 | 3617 | 5.28 [4.45, 6.27] | <.001 | 5.29 [4.46, 6.27] | <.001 | 4.97 [4.20, 5.89] | <.001 | 4.80 [4.06, 5.67] | <.001 |
|  | Model 2 | 2730 | 4.81 [3.95, 5.86] | <.001 | 5.44 [4.58, 6.47] | <.001 | 5.08 [4.28, 6.02] | <.001 | 4.89 [4.13, 5.79] | <.001 |
|  | Model 3 | 1915 | 4.81 [3.79, 6.10] | <.001 | 5.32 [4.47, 6.33] | <.001 | 4.96 [4.17, 5.90] | <.001 | 4.76 [4.00, 5.65] | <.001 |
|  | Model 4 | 1607 | 4.34 [3.32, 5.68] | <.001 | 5.01 [4.19, 5.99] | <.001 | 4.66 [3.90, 5.56] | <.001 | 4.44 [3.72, 5.30] | <.001 |
|  |  |  |  |  |  |  |  |  |  |  |
| **Harmful Drinking** | Model 1 | 3617 | 9.01 [6.63, 12.25] | <.001 | 9.00 [6.62, 12.24] | <.001 | 8.40 [6.18, 11.42] | <.001 | 7.67 [5.62, 10.45] | <.001 |
|  | Model 2 | 2730 | 8.62 [5.99, 12.41] | <.001 | 9.14 [6.71, 12.44] | <.001 | 8.45 [6.21, 11.51] | <.001 | 7.70 [5.65, 10.50] | <.001 |
|  | Model 3 | 1915 | 8.02 [5.18, 12.42] | <.001 | 8.82 [6.45, 12.04] | <.001 | 8.15 [5.97, 11.13] | <.001 | 7.49 [5.48, 10.23] | <.001 |
|  | Model 4 | 1607 | 7.06 [4.17, 11.96] | <.001 | 7.97 [5.81, 10.95] | <.001 | 7.33 [5.33, 10.07] | <.001 | 6.70 [4.88, 9.21] | <.001 |

Model 1 = unadjusted; model 2 = adjusted for sociodemographic confounders: gender, maternal education, family income, housing tenure, and social class; model 3 = additionally adjusted for parental confounders: parental depression, anxiety, alcohol use, and tobacco use; model 4 = additionally adjusted for adolescent confounders: tobacco use, cannabis use, drinking frequency, binge drinking, conduct problems, and emotional symptoms.

AD = available data; Imp#1: n = 3625; 100 imputations; Imp#2: n = 4600; 100 imputations; Imp#3: n = 9278; 200 imputations.

**Supplementary Table 5 (cont.)**

|  |  | **Available data** | | | **Imp#1** | | **Imp#2** | | **Imp#3** | |
| --- | --- | --- | --- | --- | --- | --- | --- | --- | --- | --- |
|  | **Model** | **N** | **OR [95% CI]** | **p-value** | **OR [95% CI]** | **p-value** | **OR [95% CI]** | **p-value** | **OR [95% CI]** | **p-value** |
| **Age 21** |  |  |  |  |  |  |  |  |  |  |
| **Frequent Drinking** | Model 1 | 2152 | 1.43 [1.17, 1.76] | .001 | 1.43 [1.18, 1.74] | <.001 | 1.43 [1.18, 1.73] | <.001 | 1.37 [1.13, 1.65] | .001 |
|  | Model 2 | 1678 | 1.59 [1.24, 2.02] | <.001 | 1.50 [1.23, 1.84] | <.001 | 1.51 [1.24, 1.84] | <.001 | 1.45 [1.19, 1.77] | <.001 |
|  | Model 3 | 1258 | 1.63 [1.22, 2.16] | .001 | 1.45 [1.18, 1.79] | <.001 | 1.47 [1.20, 1.80] | <.001 | 1.41 [1.15, 1.72] | .001 |
|  | Model 4 | 1084 | 1.50 [1.10, 2.06] | .012 | 1.37 [1.10, 1.69] | .005 | 1.38 [1.12, 1.70] | .002 | 1.33 [1.08, 1.64] | .008 |
|  |  |  |  |  |  |  |  |  |  |  |
| **Frequent Bingeing** | Model 1 | 2152 | 1.49 [1.21, 1.85] | <.001 | 1.51 [1.24, 1.84] | <.001 | 1.50 [1.22, 1.84] | <.001 | 1.48 [1.21, 1.81] | <.001 |
|  | Model 2 | 1678 | 1.61 [1.26, 2.06] | <.001 | 1.58 [1.29, 1.93] | <.001 | 1.56 [1.27, 1.93] | <.001 | 1.56 [1.27, 1.92] | <.001 |
|  | Model 3 | 1258 | 1.61 [1.21, 2.14] | .001 | 1.52 [1.23, 1.87] | <.001 | 1.52 [1.23, 1.88] | <.001 | 1.52 [1.23, 1.88] | <.001 |
|  | Model 4 | 1084 | 1.48 [1.08, 2.03] | .015 | 1.45 [1.17, 1.80] | .001 | 1.44 [1.16, 1.80] | .001 | 1.46 [1.16, 1.82] | .001 |
|  |  |  |  |  |  |  |  |  |  |  |
| **Hazardous Drinking** | Model 1 | 2152 | 2.15 [1.72, 2.69] | <.001 | 2.19 [1.75, 2.74] | <.001 | 2.16 [1.76, 2.65] | <.001 | 2.23 [1.83, 2.72] | <.001 |
|  | Model 2 | 1678 | 2.24 [1.73, 2.90] | <.001 | 2.28 [1.81, 2.86] | <.001 | 2.23 [1.81, 2.75] | <.001 | 2.34 [1.92, 2.86] | <.001 |
|  | Model 3 | 1258 | 2.14 [1.58, 2.90] | <.001 | 2.21 [1.75, 2.79] | <.001 | 2.18 [1.76, 2.70] | <.001 | 2.27 [1.85, 2.79] | <.001 |
|  | Model 4 | 1084 | 2.12 [1.52, 2.96] | <.001 | 2.12 [1.67, 2.69] | <.001 | 2.07 [1.66, 2.57] | <.001 | 2.15 [1.75, 2.66] | <.001 |
|  |  |  |  |  |  |  |  |  |  |  |
| **Harmful Drinking** | Model 1 | 2152 | 2.56 [1.96, 3.35] | <.001 | 2.73 [2.13, 3.51] | <.001 | 2.74 [2.14, 3.51] | <.001 | 2.63 [2.06, 3.35] | <.001 |
|  | Model 2 | 1678 | 2.75 [2.02, 3.73] | <.001 | 2.83 [2.19, 3.65] | <.001 | 2.82 [2.19, 3.62] | <.001 | 2.71 [2.12, 3.47] | <.001 |
|  | Model 3 | 1258 | 2.52 [1.76, 3.59] | <.001 | 2.70 [2.09, 3.50] | <.001 | 2.70 [2.09, 3.49] | <.001 | 2.62 [2.04, 3.36] | <.001 |
|  | Model 4 | 1084 | 2.33 [1.56, 3.48] | <.001 | 2.46 [1.88, 3.22] | <.001 | 2.46 [1.88, 3.22] | <.001 | 2.40 [1.86, 3.11] | <.001 |

Model 1 = unadjusted; model 2 = adjusted for sociodemographic confounders: gender, maternal education, family income, housing tenure, and social class; model 3 = additionally adjusted for parental confounders: parental depression, anxiety, alcohol use, and tobacco use; model 4 = additionally adjusted for adolescent confounders: tobacco use, cannabis use, drinking frequency, binge drinking, conduct problems, and emotional symptoms.

AD = available data; Imp#1: n = 3625; 100 imputations; Imp#2: n = 4600; 100 imputations; Imp#3: n = 9278; 200 imputations.

**Supplementary Table 6. Logistic regressions examining the interactions between generalized anxiety disorder and drinking to cope motives at age 18 on alcohol use at age 18 and 21.**

|  |  | **Available data** | | | **Imp 1 (n = 3625)** | | **Imp 2 (n = 4600)** | | **Imp 3 (n = 9278)** | |
| --- | --- | --- | --- | --- | --- | --- | --- | --- | --- | --- |
|  | **Model** | **N** | **OR [95% CI]** | **p-value** | **OR [95% CI]** | **p-value** | **OR [95% CI]** | **p-value** | **OR [95% CI]** | **p-value** |
| **Age 18** |  |  |  |  |  |  |  |  |  |  |
| **Frequent Drinking** | *Stratum specific* | |  |  |  |  |  |  |  |  |
|  | Low DTC | 2660 | 0.74 [0.43, 1.27] | .270 | 0.76 [0.44, 1.30] | .315 | 0.69 [0.40, 1.19] | .179 | 0.80 [0.47, 1.35] | .399 |
|  | High DTC | 792 | 1.33 [0.86, 2.06] | .204 | 1.34 [0.87, 2.06] | .188 | 1.28 [0.84, 1.96] | .258 | 1.28 [0.82, 2.00] | .270 |
|  | Interaction | 3452 | 1.80 [0.90, 3.62] | .098 | 1.77 [0.88, 3.54] | .108 | 1.86 [0.93, 3.75] | .081 | 1.61 [0.83, 3.15] | .161 |
|  |  |  |  |  |  |  |  |  |  |  |
| **Frequent Bingeing** | *Stratum specific* | |  |  |  |  |  |  |  |  |
|  | Low DTC | 2660 | 0.67 [0.31, 1.45] | .309 | 0.67 [0.31, 1.47] | .319 | 0.62 [0.29, 1.33] | .222 | 0.62 [0.29, 1.35] | .231 |
|  | High DTC | 792 | 1.11 [0.69, 1.78] | .678 | 1.15 [0.72, 1.84] | .557 | 1.10 [0.69, 1.77] | .691 | 1.13 [0.71, 1.79] | .614 |
|  | Interaction | 3452 | 1.66 [0.67, 4.12] | .278 | 1.71 [0.69, 4.25] | .248 | 1.77 [0.71, 4.40] | .218 | 1.81 [0.74, 4.44] | .197 |
|  |  |  |  |  |  |  |  |  |  |  |
| **Hazardous Drinking** | *Stratum specific* | |  |  |  |  |  |  |  |  |
|  | Low DTC | 2660 | 0.95 [0.62, 1.45] | .810 | 0.96 [0.63, 1.47] | .850 | 0.94 [0.61, 1.42] | .756 | 0.99 [0.65, 1.50] | .959 |
|  | High DTC | 792 | 0.91 [0.56, 1.48] | .701 | 0.92 [0.57, 1.49] | .737 | 0.89 [0.55, 1.42] | .616 | 0.90 [0.57, 1.43] | .664 |
|  | Interaction | 3452 | 0.96 [0.50, 1.82] | .896 | 0.96 [0.50, 1.82] | .899 | 0.95 [0.49, 1.82] | .869 | 0.91 [0.49, 1.72] | .780 |
|  |  |  |  |  |  |  |  |  |  |  |
| **Harmful Drinking** | *Stratum specific* | |  |  |  |  |  |  |  |  |
|  | Low DTC | 2660 | 1.30 [0.40, 4.21] | .664 | 1.30 [0.40, 4.23] | .659 | 1.30 [0.41, 4.17] | .658 | 1.34 [0.43, 4.11] | .613 |
|  | High DTC | 792 | 1.10 [0.63, 1.93] | .737 | 1.12 [0.64, 1.96] | .693 | 1.09 [0.62, 1.91] | .766 | 1.11 [0.64, 1.95] | .708 |
|  | Interaction | 3452 | 0.85 [0.23, 3.13] | .805 | 0.86 [0.23, 3.17] | .820 | 0.84 [0.23, 3.07] | .788 | 0.83 [0.23, 3.02] | .780 |

Unadjusted model. Stratified analysis: associations of generalized anxiety disorder at age 18 with alcohol use outcomes at age 18 and 21 in each stratum of drinking to cope motives. Interaction term: interaction of GAD x DTC at age 18 on alcohol use outcomes at age 18 and 21.

AD = available data; Imp#1: n = 3625; 100 imputations; Imp#2: n = 4600; 100 imputations; Imp#3: n = 9278; 200 imputations.

**Supplementary Table 6 (cont.)**

|  |  | **Available data** | | | **Imp#1** | | **Imp#2** | | **Imp#3** | |
| --- | --- | --- | --- | --- | --- | --- | --- | --- | --- | --- |
|  | **Model** | **N** | **OR [95% CI]** | **p-value** | **OR [95% CI]** | **p-value** | **OR [95% CI]** | **p-value** | **OR [95% CI]** | **p-value** |
| **Age 21** |  |  |  |  |  |  |  |  |  |  |
| **Frequent Drinking** | *Stratum specific* | |  |  |  |  |  |  |  |  |
|  | Low DTC | 1621 | 1.19 [0.73, 1.94] | .493 | 1.16 [0.71, 1.89] | .550 | 1.17 [0.75, 1.84] | .488 | 1.16 [0.75, 1.78] | .502 |
|  | High DTC | 444 | 1.19 [0.68, 2.09] | .542 | 1.17 [0.68, 2.00] | .578 | 1.23 [0.73, 2.06] | .432 | 1.30 [0.78, 2.17] | .318 |
|  | Interaction | 2065 | 1.00 [0.48, 2.11] | .994 | 1.00 [0.49, 2.04] | .991 | 1.05 [0.53, 2.08] | .895 | 1.12 [0.56, 2.23] | .745 |
|  |  |  |  |  |  |  |  |  |  |  |
| **Frequent Bingeing** | *Stratum specific* | |  |  |  |  |  |  |  |  |
|  | Low DTC | 1621 | 0.89 [0.52, 1.53] | .683 | 0.89 [0.52, 1.50] | .651 | 0.86 [0.53, 1.41] | .551 | 0.88 [0.53, 1.45] | .609 |
|  | High DTC | 444 | 0.85 [0.47, 1.51] | .570 | 0.91 [0.51, 1.61] | .736 | 0.88 [0.52, 1.49] | .636 | 0.90 [0.52, 1.57] | .714 |
|  | Interaction | 2065 | 0.95 [0.43, 2.09] | .892 | 1.02 [0.46, 2.27] | .955 | 1.02 [0.50, 2.09] | .952 | 1.03 [0.51, 2.09] | .936 |
|  |  |  |  |  |  |  |  |  |  |  |
| **Hazardous Drinking** | *Stratum specific* | |  |  |  |  |  |  |  |  |
|  | Low DTC | 1621 | 1.10 [0.68, 1.80] | .693 | 1.01 [0.64, 1.59] | .966 | 0.92 [0.59, 1.45] | .728 | 0.96 [0.61, 1.51] | .869 |
|  | High DTC | 444 | 0.93 [0.50, 1.73] | .813 | 0.96 [0.53, 1.75] | .905 | 0.90 [0.50, 1.62] | .719 | 0.92 [0.53, 1.60] | .766 |
|  | Interaction | 2065 | 0.84 [0.38, 1.85] | .667 | 0.95 [0.45, 2.01] | .903 | 0.97 [0.46, 2.04] | .942 | 0.96 [0.44, 2.07] | .907 |
|  |  |  |  |  |  |  |  |  |  |  |
| **Harmful Drinking** | *Stratum specific* | |  |  |  |  |  |  |  |  |
|  | Low DTC | 1621 | 1.54 [0.77, 3.08] | .218 | 1.56 [0.78, 3.11] | .208 | 1.63 [0.87, 3.07] | .126 | 1.73 [0.90, 3.32] | .099 |
|  | High DTC | 444 | 1.12 [0.58, 2.14] | .733 | 1.08 [0.59, 2.00] | .798 | 1.09 [0.62, 1.92] | .775 | 1.09 [0.59, 2.02] | .772 |
|  | Interaction | 2065 | 0.73 [0.28, 1.87] | .507 | 0.69 [0.28, 1.71] | .428 | 0.66 [0.28, 1.59] | .357 | 0.63 [0.26, 1.56] | .321 |

Unadjusted model. Stratified analysis: associations of generalized anxiety disorder at age 18 with alcohol use outcomes at age 18 and 21 in each stratum of drinking to cope motives. Interaction term: interaction of GAD x DTC at age 18 on alcohol use outcomes at age 18 and 21.

AD = available data; Imp#1: n = 3625; 100 imputations; Imp#2: n = 4600; 100 imputations; Imp#3: n = 9278; 200 imputations.

**Supplementary Table 7. Logistic regressions examining the associations of generalized anxiety disorder at age 18 with alcohol use at age 18 and 21, including non-drinkers at age 18 (available data only).**

|  |  |  | **Frequent Drinking** | | **Frequent Bingeing** | | **Hazardous Drinking** | | **Harmful Drinking** | |
| --- | --- | --- | --- | --- | --- | --- | --- | --- | --- | --- |
|  | **Model** | **N** | **OR [95% CI]** | **p-value** | **OR [95% CI]** | **p-value** | **OR [95% CI]** | **p-value** | **OR [95% CI]** | **p-value** |
| **Age 18** |  |  |  |  |  |  |  |  |  |  |
|  | Model 1 | 3727 | 1.40 [1.03, 1.91] | .031 | 1.41 [0.97, 2.05] | .071 | 1.43 [1.08, 1.90] | .012 | 2.00 [1.23, 3.24] | .005 |
|  | Model 2 | 2798 | 1.69 [1.19, 2.41] | .004 | 1.65 [1.08, 2.54] | .022 | 1.58 [1.14, 2.20] | .006 | 2.46 [1.41, 4.29] | .002 |
|  | Model 3 | 1957 | 1.73 [1.12, 2.69] | .014 | 1.78 [1.05, 3.03] | .033 | 1.95 [1.30, 2.94] | .001 | 3.48 [1.87, 6.48] | <.001 |
|  | Model 4 | 1641 | 1.62 [0.97, 2.72] | .065 | 1.64 [0.87, 3.11] | .129 | 1.82 [1.14, 2.92] | .013 | 3.96 [1.83, 8.58] | <.001 |
| **Age 21** |  |  |  |  |  |  |  |  |  |  |
|  | Model 1 | 2511 | 1.24 [0.89, 1.73] | .205 | 0.94 [0.65, 1.35] | .724 | 1.13 [0.81, 1.58] | .467 | 1.68 [1.09, 2.58] | .018 |
|  | Model 2 | 1936 | 1.27 [0.85, 1.89] | .236 | 0.87 [0.56, 1.34] | .522 | 1.11 [0.75, 1.64] | .597 | 1.48 [0.87, 2.52] | .148 |
|  | Model 3 | 1445 | 1.67 [1.03, 2.70] | .039 | 0.94 [0.56, 1.57] | .817 | 1.59 [0.97, 2.60] | .065 | 1.49 [0.80, 2.78] | .208 |
|  | Model 4 | 1224 | 1.35 [0.77, 2.36] | .289 | 0.69 [0.37, 1.28] | .238 | 1.36 [0.79, 2.36] | .271 | 1.28 [0.60, 2.74] | .526 |

Model 1 = unadjusted; model 2 = adjusted for sociodemographic confounders: gender, maternal education, family income, housing tenure, and social class; model 3 = additionally adjusted for parental confounders: parental depression, anxiety, alcohol use, and tobacco use; model 4 = additionally adjusted for adolescent confounders: tobacco use, cannabis use, drinking frequency, binge drinking, conduct problems, and emotional symptoms.

**Supplementary Table 8. Logistic regressions examining the associations of generalized anxiety disorder at age 18 with alcohol use at age 18 and 21, with an alternative control group (available data only).**

|  |  |  | **Frequent Drinking** | | **Frequent Bingeing** | | **Hazardous Drinking** | | **Harmful Drinking** | |
| --- | --- | --- | --- | --- | --- | --- | --- | --- | --- | --- |
|  | **Model** | **N** | **OR [95% CI]** | **p-value** | **OR [95% CI]** | **p-value** | **OR [95% CI]** | **p-value** | **OR [95% CI]** | **p-value** |
| **Age 18** |  |  |  |  |  |  |  |  |  |  |
|  | Model 1 | 3407 | 1.42 [1.05, 1.94] | .025 | 1.44 [0.99, 2.09] | .057 | 1.49 [1.13, 1.98] | .005 | 2.17 [1.33, 3.54] | .002 |
|  | Model 2 | 2560 | 1.74 [1.22, 2.49] | .002 | 1.71 [1.11, 2.64] | .015 | 1.69 [1.21, 2.34] | .002 | 2.75 [1.57, 4.84] | <.001 |
|  | Model 3 | 1805 | 1.80 [1.16, 2.79] | .009 | 1.81 [1.06, 3.10] | .029 | 2.09 [1.39, 3.14] | <.001 | 3.82 [2.03, 7.19] | <.001 |
|  | Model 4 | 1525 | 1.71 [1.02, 2.87] | .043 | 1.70 [0.90, 3.25] | .105 | 1.94 [1.21, 3.12] | .006 | 4.35 [1.99, 9.52] | <.001 |
| **Age 21** |  |  |  |  |  |  |  |  |  |  |
|  | Model 1 | 2306 | 1.20 [0.86, 1.68] | .288 | 0.92 [0.64, 1.32] | .645 | 1.15 [0.82, 1.60] | .429 | 1.69 [1.10, 2.60] | .017 |
|  | Model 2 | 1778 | 1.22 [0.82, 1.82] | .322 | 0.86 [0.55, 1.32] | .482 | 1.12 [0.75, 1.65] | .582 | 1.51 [0.88, 2.57] | .134 |
|  | Model 3 | 1333 | 1.61 [0.99, 2.62] | .053 | 0.94 [0.56, 1.58] | .821 | 1.63 [0.99, 2.67] | .054 | 1.50 [0.80, 2.80] | .207 |
|  | Model 4 | 1128 | 1.30 [0.74, 2.28] | .356 | 0.68 [0.37, 1.27] | .224 | 1.36 [0.78, 2.37] | .276 | 1.25 [0.58, 2.68] | .566 |

Alternative control group: individuals with no GAD or any other type of anxiety or depression. Model 1 = unadjusted; model 2 = adjusted for sociodemographic confounders: gender, maternal education, family income, housing tenure, and social class; model 3 = additionally adjusted for parental confounders: parental depression, anxiety, alcohol use, and tobacco use; model 4 = additionally adjusted for adolescent confounders: tobacco use, cannabis use, drinking frequency, binge drinking, conduct problems, and emotional symptoms.
